# Mesenchymal stromal cells-derived matrix Gla protein contribute to the alleviation of experimental colitis

**DOI:** 10.1038/s41419-018-0734-3

**Published:** 2018-06-07

**Authors:** Yuan Feng, Yan Liao, Weijun Huang, Xingqiang Lai, Jing Luo, Cong Du, Junyi Lin, Zhongyuan Zhang, Dongbo Qiu, Qiuli Liu, Huiyong Shen, Andy Peng Xiang, Qi Zhang

**Affiliations:** 1 Guangdong Provincial Key Laboratory of Liver Disease Research, The Third Affiliated Hospital, Sun Yat-sen University, Guangzhou, China; 2 Cell-gene Therapy Translational Medicine Research Center, The Third Affiliated Hospital, Sun Yat-sen University, Guangzhou, China; 3 Center for Stem Cell Biology and Tissue Engineering, Key Laboratory for Stem Cells and Tissue Engineering, Ministry of Education, Sun Yat-Sen University, Guangzhou, China; 4 Department of Rehabilitation Medicine, The Third Affiliated Hospital, Sun Yat-Sen University, Guangzhou, China; 5 Zhongshan School of Medicine, Sun Yat-sen University, Guangzhou, China; 6 Biotherapy Center, The Third Affiliated Hospital, Sun Yat-sen University, Guangzhou, China; 7 Department of Orthopedics, Sun Yat-sen Memorial Hospital, Sun Yat-sen University, Guangzhou, People’s Republic of China

## Abstract

Crohn’s disease (CD) is a chronic inflammatory bowel disease that is difficult to treat. However, previous preclinical and clinical studies have shown that mesenchymal stromal cells (MSCs) are a promising therapeutic approach, whereas the exact underlying molecular mechanisms of MSCs in treating CD remain unclear. Furthermore, the heterogeneity of MSCs, as well as the in vivo microenvironments may influence the therapeutic efficacy. In our previous study, we found that a subpopulation of mouse MSCs with a high expression of matrix Gla protein (MGP), one of the members of vitamin K-dependent protein family, possessed better immunoregulatory properties. Therefore, in this study we investigate whether the abundant MSCs-derived MGP participate in the therapeutic mechanisms for MSCs treating CD. Obvious suppression of cell proliferation and cytokine production in T cells were observed in vitro through MSCs-derived MGP. Moreover, MGP alleviated the clinical and histopathological severity of colonic inflammation in mouse experimental colitis models to a remarkable degree. Our results indicate that MGP might be a novel important mediator of MSCs-mediated immunomodulation in treating CD.

## Introduction

Crohn’s disease (CD) is a multifactorial chronic relapsing disease of the colon and small intestine, triggered by a loss of balance between pro-inflammatory T cells and regulatory T lymphocytes, which results in the production of various pro-inflammatory cytokines and lymphocytes infiltrating the gut^1–4^. Patients with CD suffer abdominal pain, diarrhea, weight loss, and fever, affecting the quality of life of sufferers^4^, but currently there is no effective treatment. Therefore, a new therapeutic strategy is urgently needed. During the past two decades, therapies based on mesenchymal stem cells (MSCs) have attracted great interest as new treatments in a range of refractory or incurable diseases—including a variety of inflammatory and autoimmune diseases. This is due to their self-renewal capacity, multipotency, and potent immunomodulatory effects. MSCs have showed their potential in treating CD in preclinical experiments and a few clinical trials^5,6^. However, the underlying molecular mechanism of MSCs in treating CD remains largely unknown.

In order to promote the clinical application of MSCs in treating CD, it is necessary to characterize the subpopulations of MSCs that possess significant stable curative effects in the disease microenvironment, as well as delineating the key factors mediating this immunoregulatory function. In our study^7^, it was noticed that one of our mouse bone marrow MSCs subpopulations possessed a higher immunosuppressive ability and express high levels of VKDPs-related genes, which are a group of proteins undergoing vitamin K-dependent post-translational processing.

Multiple studies have revealed that vitamin K might be important to the progress of CD^8–13^. In view of the fact that the VKDPs family act as a functional element downstream of vitamin K signaling, it is suggested that VKDPs may be related to CD development. Although the coagulation factors are the most well-known VKDPs, there are many others with important physiologic roles related to bone mineralization, arterial calcification, apoptosis, phagocytosis, growth control, chemotaxis, and signal transduction^14^. Recent advances have also suggested their role in the immunomodulatory functions^15–17^. In the previous study^7^, we reported that MSC4, one of the subpopulations in the MSC family, possesses trilineage differentiation abilities, exhibits superior immunomodulation ability, and expresses the highest levels of matrix Gla protein (MGP) in the VKDPs family.

MGP is a secreted protein and acts as a bone morphogenetic protein signaling inhibitor and has high affinity for calcium ions^18^. Recent studies showed its key role in the protection of atherosclerosis and angiosteosis^19–21^, and indicated that it might be relevant to inflammation^20,22^. Hence, we hypothesize that high-expressed MGP might contribute to the immunomodulatory functions of MSCs, and if MSCs with abundant MGP could be an effective CD therapy.

## Results

### MGP is highly expressed in a subpopulation of mouse bone marrow MSCs with superior immunomodulatory ability

**Fig. 1:**
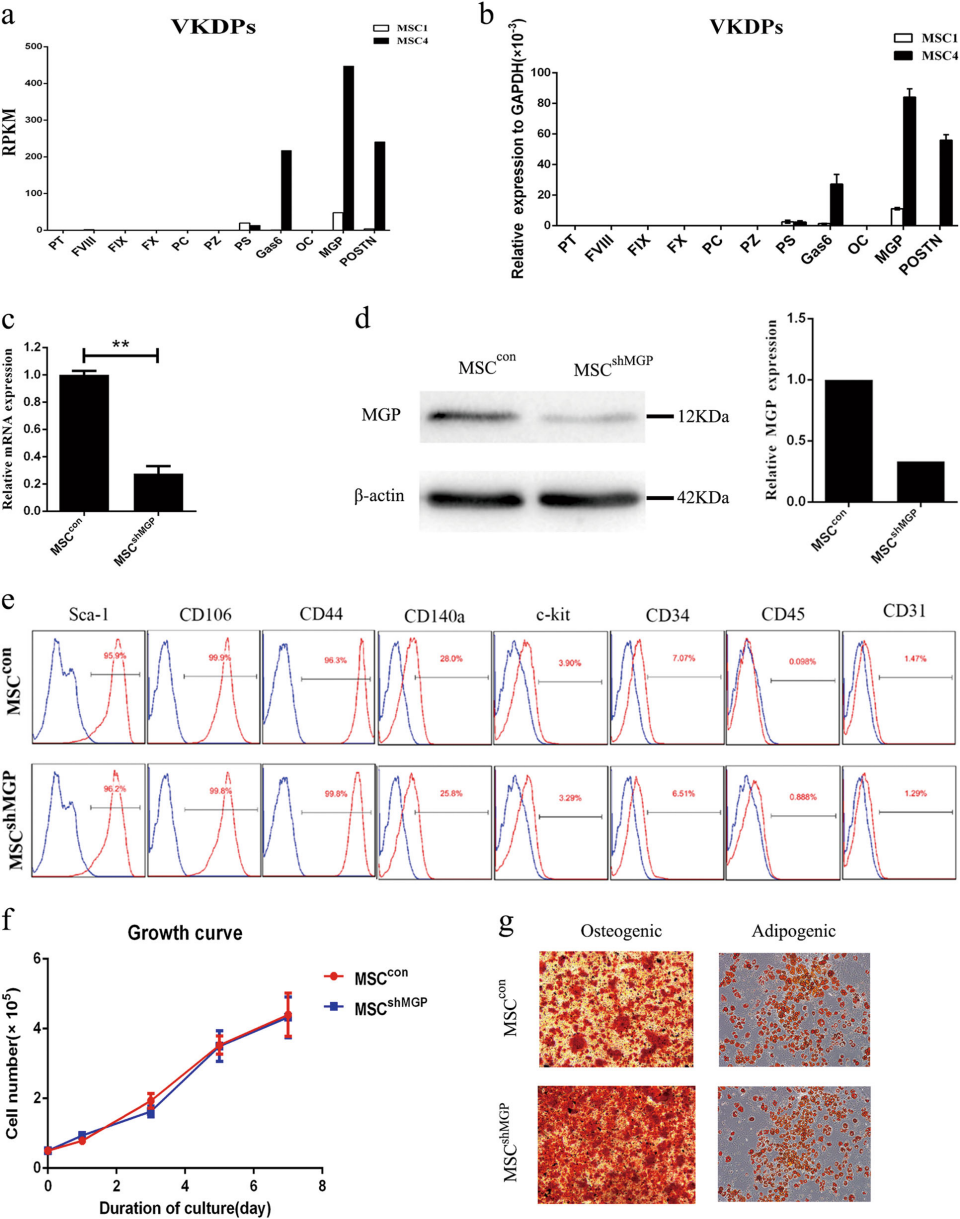
MGP is the highest expression member of VKDPs in mouse MSCs, the properties of which does not alter when MGP knockdown. **a** RNA-seq analysis about the expression of VKDPs in MSC1 and MSC4. **b** The relative mRNA expression levels of VKDPs were analyzed by qPCR. The results were normalized with respect to the expression of GAPDH. **c** The interfering efficiency of shRNA technique on the expression of MGP was assessed at the RNA level. The expression of MGP in MSC^con^ was regarded as 1. **d** The interfering efficiency of shRNA technique on the expression of MGP was assessed at the protein level. The expression of β-actin was used as a control. **e** Flow cytometry was adopted to analyze the expression of surface markers. **f** Growth curves of MSC^con^ and MSC^shMGP^ were assessed by cell counting for 7 days. Three replicates were performed at each time point. **g** Adipogenic and osteogenic differentiations of MSC^con^ and MSC^shMGP^. Scale bar = 100 μm. Data are shown as mean ± SEM (*n* = 3). **P < 0.01. SEM, standard error of mean

Our previous study^7^ found that a subpopulation MSC4 possessed trilineage differentiation abilities and exhibited better immunoregulatory properties, whereas the other subpopulation MSC1 possessed particularly poor immunoregulatory abilities. Further RNA-seq analysis screened out the highly expressed genes in MSC4. Compared with MSC1, several members of the VKDPs family were highly expressed in MSC4, among which MGP was the most abundant gene (Fig. 1a). We detected the expressions of VKDPs using quantitative polymerase chain reaction (qPCR) and confirmed that MGP was the most highly expressed member in MSCs. Specifically, the expression of other VKDPs members, including protein S (PS), growth arrest-specific protein 6 (Gas6), osteocalcin (OC), and periostin (POSTN) were lower compared with MGP, and the expressions of prothrombin, factor VIII (F VIII), FIX, FX, protein Z, and protein C (PC) were extremely weak (Fig. 1b).

To investigate the effect of MGP on the immunomodulatory properties of MSC, we constructed lentivirus carrying short hairpin RNA (shRNA) targeting MGP in MSCs (MSC^shMGP^) and analyzed the knockdown efficiency by qPCR and western blotting. The qPCR results showed that ~ 70% of MGP expression was suppressed compared with MSCs transduced with insert-free (empty) lentiviral construct (MSC^con^) (Fig. 1c). Western blotting analysis was also consistent with the result of qPCR (Fig. 1d). The secretion levels of MSCs-derived MGP were analyzed by enzyme-linked immunosorbent assay (ELISA). The results showed that MSCs-derived MGP could continuously secrete into supernatant among 5 days (Supplementary Figure S1a) after transfection, and the secretion was decreased significantly by shRNA interference on day 3 after treatment (Supplementary Figure S1b).

To investigate whether MGP knockdown could affect the characteristics of the MSCs, we used flow cytometry to analyze the cell surface markers, including Sca-1, CD106, CD44, CD140a, c-kit, CD34, CD45, and CD31, and found that MSC^con^ and MSC^shMGP^ shared the same expression profile (Fig. 1e), which indicated that MGP knockdown in MSCs did not change their phenotype. Moreover, Cell Counting Kit-8 (CCK-8, Supplementary Figure S2a) and direct cell counting (Fig. 1f) indicated that MGP knockdown did not change the proliferation efficiency of MSCs. Cell apoptosis was detected by flow cytometric analysis through staining Annexin V and PI dye and by direct cell counting after trypan blue staining (Fig. S2b–c). The results suggested the MGP downregulation did not affect cellular viabilities. In addition, we cultured MSC^con^ and MSC^shMGP^ cells under conditions that promote their differentiation into osteogenic and adipogenic cells. As confirmed by Alizarin Red S and Oil Red O staining, respectively, the MSC^shMGP^ cells retained the differentiation capacity compared with MSC^con^ (Fig. 1g). Taken together, MGP is the highest expressed member of the VKDPs family in mouse MSCs, and MGP knockdown did not change the related properties of MSCs.

### MSCs-derived MGP suppresses the proliferation of activated T cells in vitro

**Fig. 2:**
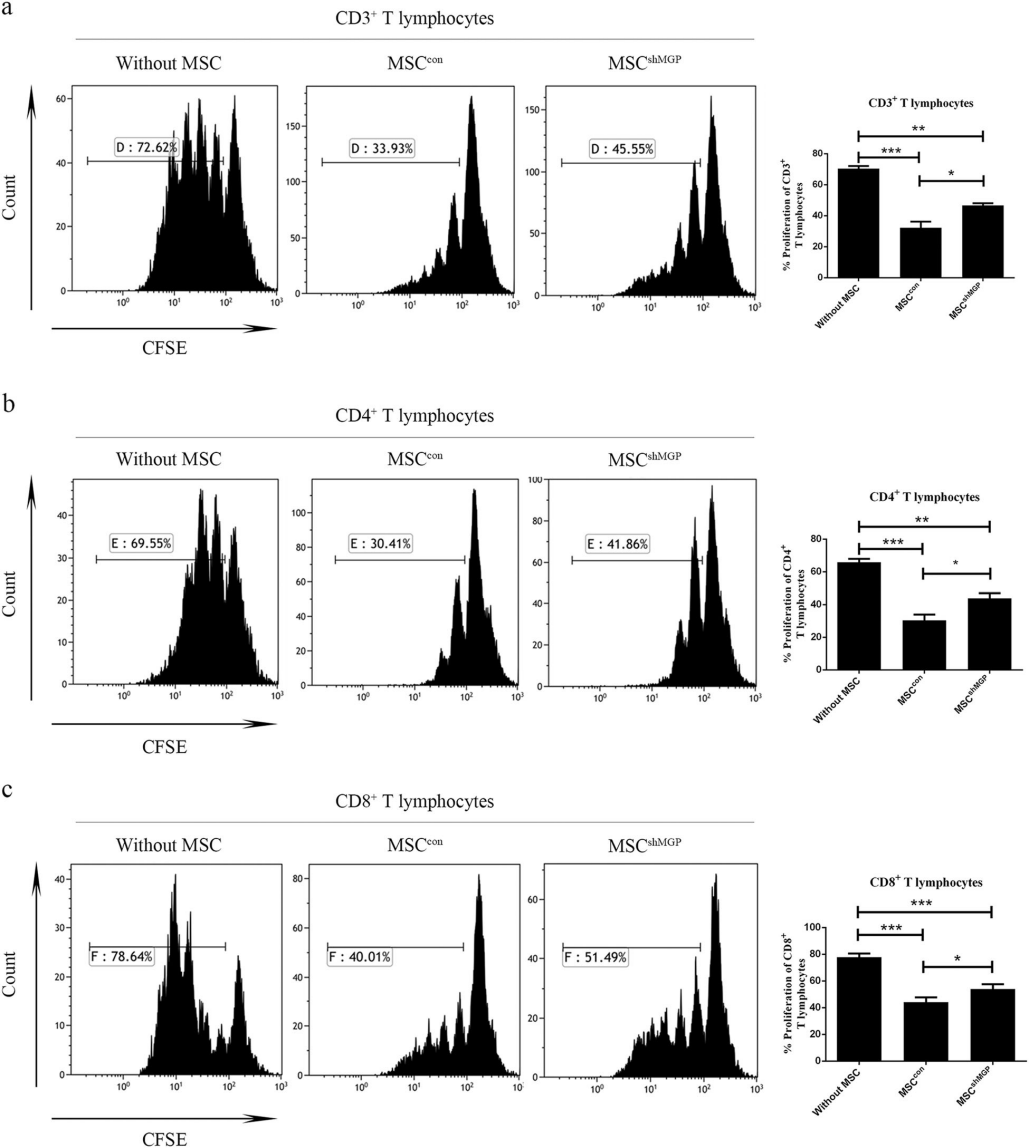
Mouse MSCs-derived MGP inhibits the proliferation of activated T cells in vitro (verified by RNA interference). The proliferation levels of mouse CD3^+^ T cells **a**, CD4^+^ T cells **b**, and CD8^+^ T cells **c** were analyzed by flow cytometry; the change of CFSE fluorescence intensity indicates the growth ratio. Data are shown as mean ± SEM (*n* = 3). *P < 0.05, **P < 0.01, ***P < 0.001, and n.s. means no significant

To verify whether MGP contributes to the immunomodulatory properties of MSCs, the anti-proliferation effects of MSC^con^ and MSC^shMGP^ were analyzed on activated T cells. As shown by in vitro co-culture experiments, the proliferation percentage of T-cell populations in MSC^shMGP^ was significantly higher compared with MSC^con^ for the population analysis in CD3^+^ (46.50 ± 2.68% versus 32.24 ± 8.02%, Fig. 2a), CD4^+^ (43.93 ± 6.70% versus 30.46 ± 6.93%, Fig. 2b), and CD8^+^ (54.02 ± 3.52% versus 44.13 ± 3.57%, Fig. 2c) T cells.

To confirm these results further, we used two small guide RNAs (sgRNAs) of different sequences targeting the MGP and constructed the MGP-knockout MSCs with a CRISPR/Cas9 system (Supplementary Figure S3a). Both of the two sgRNAs specifically targeting MGP caused a significant MGP silencing at protein levels in the transduced MSCs (Supplementary Figure S3b). The MGP silencing in MSCs also partially restored the proliferation of T cells in CD3^+^ (sg1: 51.15 ± 4.62%; sg2: 62.69 ± 2.56% versus 37.26 ± 8.57%, Supplementary Figure S4a), CD4^+^ (sg1: 49.16 ± 4.23%; sg2: 53.81 ± 1.69% versus 29.54 ± 8.03%, Supplementary Figure S4b) and CD8^+^ (sg1: 69.56 ± 5.72%; sg2: 69.86 ± 5.64% versus 50.18 ± 11.06%, Supplementary Figure S4c).

Since MGP is a secreted protein, we collected MSCs-derived supernatant as conditioned medium (MSC-CM) to examine its effect on the proliferation of activated T cells. As shown by in vitro co-culture experiments, the proliferation percentage of T-cell populations in MSC^shMGP^-CM was higher when compared with MSC^con^-CM, including CD3^+^ (66.41 ± 2.60% versus 58.90 ± 4.57%, Supplementary Figure S5a), CD4^+^ (57.01 ± 3.80% versus 45.41 ± 3.12%, Supplementary Figure S5b), and CD8^+^ (72.95 ± 1.10% versus 66.95 ± 4.18%, Supplementary Figure S5c) T cells. The results indicated MSCs-secreted MGP could suppress the proliferation of activated T cells through a paracrine manner.

From the above experiments, our data showed that MGP play an important role in the immunomodulatory properties of MSCs.

### MSCs-derived MGP suppresses the cytokine production of activated T cells

MSCs are known to inhibit the secretion of pro-inflammatory cytokines by T cells^23^. To verify whether MSCs-MGP contributes to this function of MSCs, we analyzed the suppression effect on tumor necrosis factor-α (TNF-α) and interferon-γ (IFN-γ) expression of T cells treated with MSC^con^ and MSC^shMGP^. Notably it was shown that, using flow cytometry analysis, T cells had a high expression of TNF-α and IFN-γ after stimulation with anti-CD3/CD8 antibody, and MSC^con^ could inhibit the expression of pro-inflammatory cytokines when co-cultured with T cells.

**Fig. 3:**
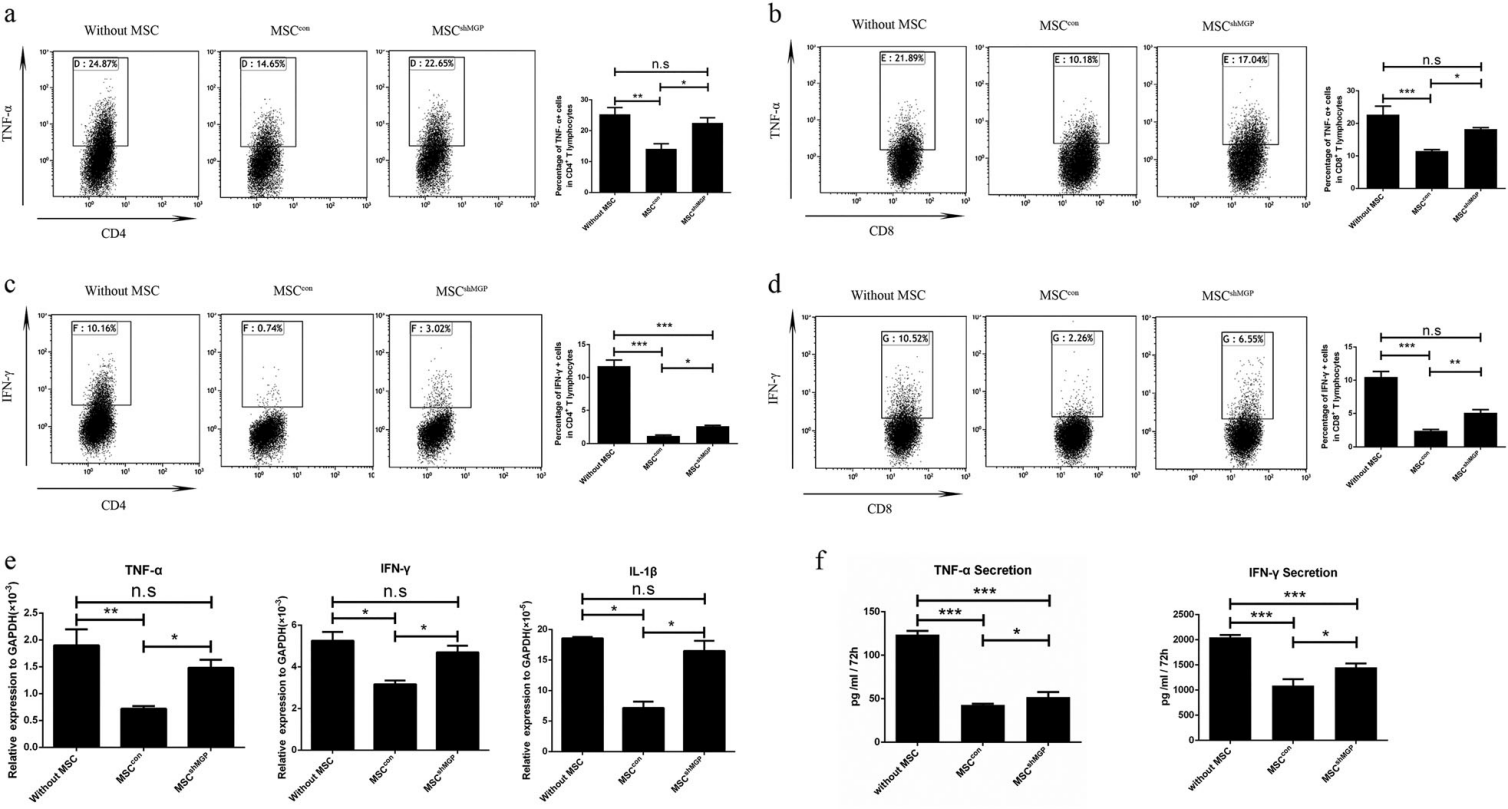
Mouse MSCs-derived MGP downregulates the cytokine production of activated T cells (verified by RNA interference). Flow cytometry was adopted to analyze the expression levels of TNF-α and IFN-γ in CD4^+^ T cells (**a**) and (**c**), respectively, and CD8^+^ T cells (**b**) and (**d**), respectively, after 3 days of co-culture with MSCs. **e** The expression levels of pro-inflammation cytokines (TNF-α, IFN-γ, and IL-1β) were analyzed at the mRNA level. **f** The secretion levels of pro-inflammation cytokines (TNF-α and IFN-γ) were analyzed by ELISA. Data are shown as mean ± SEM (*n* = 3). *P < 0.05, **P < 0.01, ***P < 0.001, and n.s. means no significant

The expression of pro-inflammatory cytokines was significantly reduced by MSC^con^ compared with MSC^shMGP^ in TNF-α (13.92 ± 4.11% versus 23.30 ± 1.65%) and IFN-γ (1.08 ± 0.24% versus 2.62 ± 0.39%) of CD4^+^ T cells (Fig. 3a,c), TNF-α (11.47 ± 0.99% versus 18.18 ± 0.96%) and IFN-γ (2.25 ± 0.67% versus 5.07 ± 1.53%) of CD8^+^ T cells (Fig. 3b,d). Similar phenomena were also observed through MGP silencing in MSCs through the small guide RNA (sgRNA) approach (Supplementary Figure S6).

We further confirmed the results by qPCR (Fig. 3e) and ELISA (Fig. 3f), which suggested that the MGP knockdown in MSCs restored the TNF-α and IFN-γ production of T cells. Similar phenomena were observed through MGP silencing in MSCs using the sgRNA approach (Supplementary Figure S7).

Meanwhile, we detected the effect of MSC-CM on the cytokine secretion of activated T cells. The results showed that the expression of TNF-α and IFN-γ were reduced by MSC^con^-CM, and this effect of MSC^shMGP^-CM was much weaker (Supplementary Figure S5d–g). These results indicated that MGP is a key mediator in immunomodulatory function of MSCs by inhibiting the expression of pro-inflammatory cytokines from activated T cells.

### MSC-derived MGP shows less effect on T cells apoptosis and Treg differentiation

Previous studies have revealed that MSCs are able to induce apoptosis of activated T cells^24^. Therefore, we analyzed the potential role of MSCs-derived MGP in inducing apoptosis of activated T cells in a MSCs/CD3^+^ T cells co-culture experiment. We found there was no difference in the proportion of Annexin V^+^/PI^+^ cells in CD3^+^ T cells between the MSC^con^ and MSC^shMGP^ groups (Supplementary Figure S8a). The results indicate that MSCs-derived MGP may not change the apoptosis of activated T cells.

Previous studies have reported that MSCs play an immunomodulatory role by promoting Treg’s differentiation^23,25,26^. In this study, the influence of MSCs on the proportion of Treg cells was analyzed. The percentage of Treg cells in MSC/CD3^+^ T cells co-culture experiments were measured, and it was found that there was an increased proportion of CD4^+^CD25^+^Foxp3^+^ Tregs in both MSC^con^ and MSC^shMGP^ groups, whereas no difference was observed between these two groups, which suggested that MSCs-derived MGP may not play a role in the Treg’s differentiation (Supplementary Figure S8b).

### MSC-derived MGP alleviates TNBS-induced experimental colitis in vivo

To verify whether MGP contributes to the therapeutic effect of MSCs in vivo, we established the experimental colitis mouse model with 2,4,6-trinitrobenzene sulfonic acid (TNBS), which shows symptoms similar to clinical CD^27,28^. MSCs (MSC^con^ or MSC^shMGP^) were injected intraperitoneally (i.p.). The negative control group was injected intraperitoneally with saline.

**Fig. 4:**
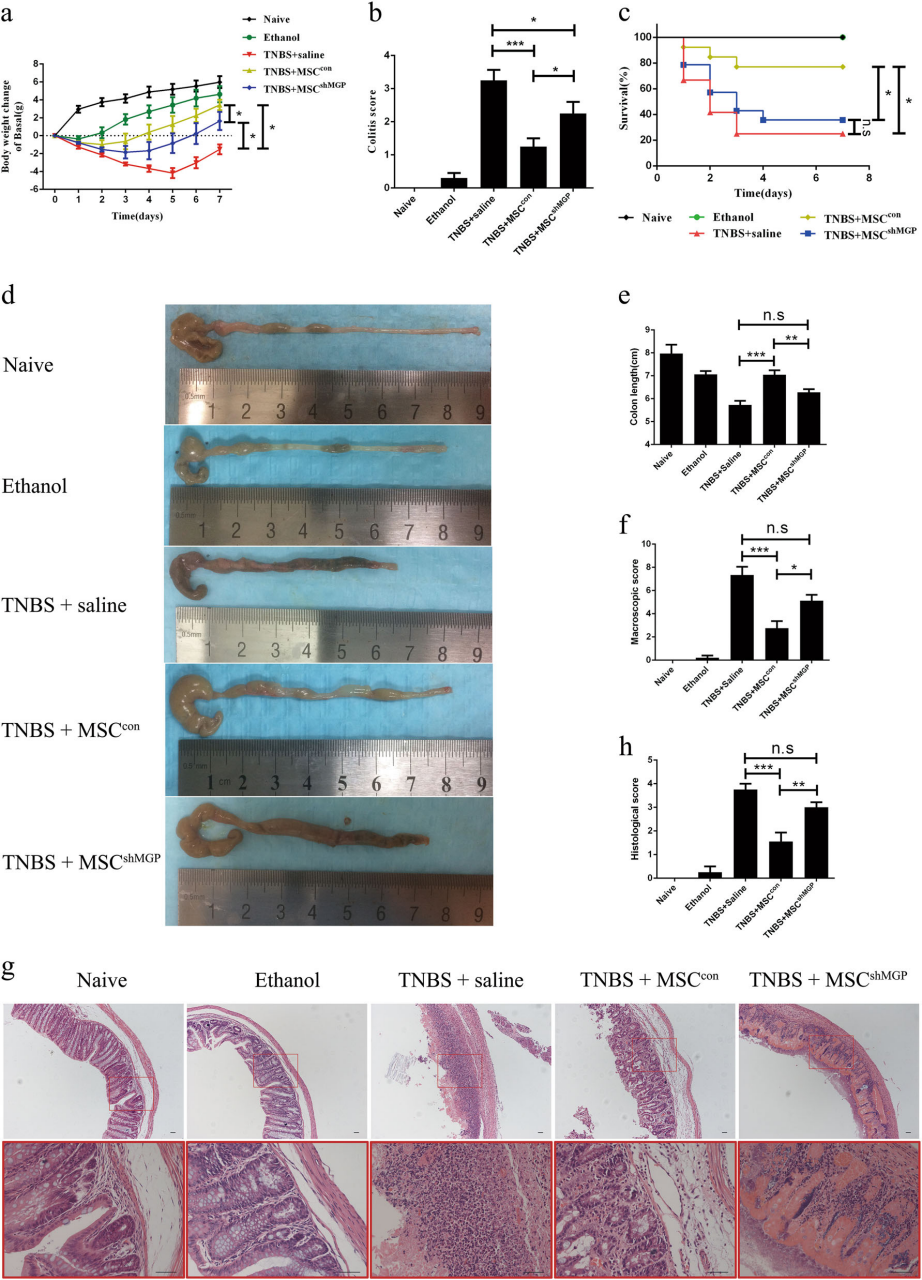
MSC-derived MGP mitigates TNBS-induced experimental colitis. Clinical evaluation of the colitis was monitored (*n* = 10 per group) based on body weight (**a**), colitis score (**b**), and survival rate **(c)**. Colons were examined with respect to general form, length (**d**, **e**), and macroscopic scores (**f**) 3 days after TNBS intracolonic administration (*n* = 4 per group). Histopathologic analysis (H&E staining and histological score) (**g**, **h**) were determined 3 days after transplantation of the cells (*n* = 3 per group). Scale bar = 40 μm. Data are shown as mean ± SEM. *P < 0.05, **P < 0.01, ***P < 0.001, and n.s. means no significant

Our data show that the body weight of mice that suffered colitis increased obviously 3 days after MSC^con^ treatment compared with a minor increase 3 days after MSC^shMGP^ injection (Fig. 4a). The colitis score was evaluated by hair condition and degree of diarrhea. As shown in Fig. 4b, the activity was significantly relieved by MSC^con^ treatment compared with MSC^shMGP^ (1.25 ± 0.87 points versus 2.25 ± 1.22 points). A similar tendency was also observed in the survival rate (76.92% versus 35.71%, Fig. 4c), colon length (7.04 ± 0.60 cm versus 6.28 ± 0.46 cm, Fig. 4d, e), and macroscopic score according to the degree of edema and hyperemia (2.75 ± 1.75 points versus 5.12 ± 1.46 points, Fig. 4f). Hematoxylin–eosin staining (Fig. 4g) also indicated that there was a difference in the status of inflammation cell infiltration that corresponded with the histological score (1.56 ± 1.13 points versus 3.00 ± 0.74 points, Fig. 4h). Taking these together, it is obvious that MSC-derived MGP is significant in alleviating symptoms of experimental colitis in vivo.

### MSC-derived MGP alleviates T cells infiltrating and suppresses the expression of pro-inflammatory cytokines in colon tissues of mice subjected to experimental colitis

**Fig. 5:**
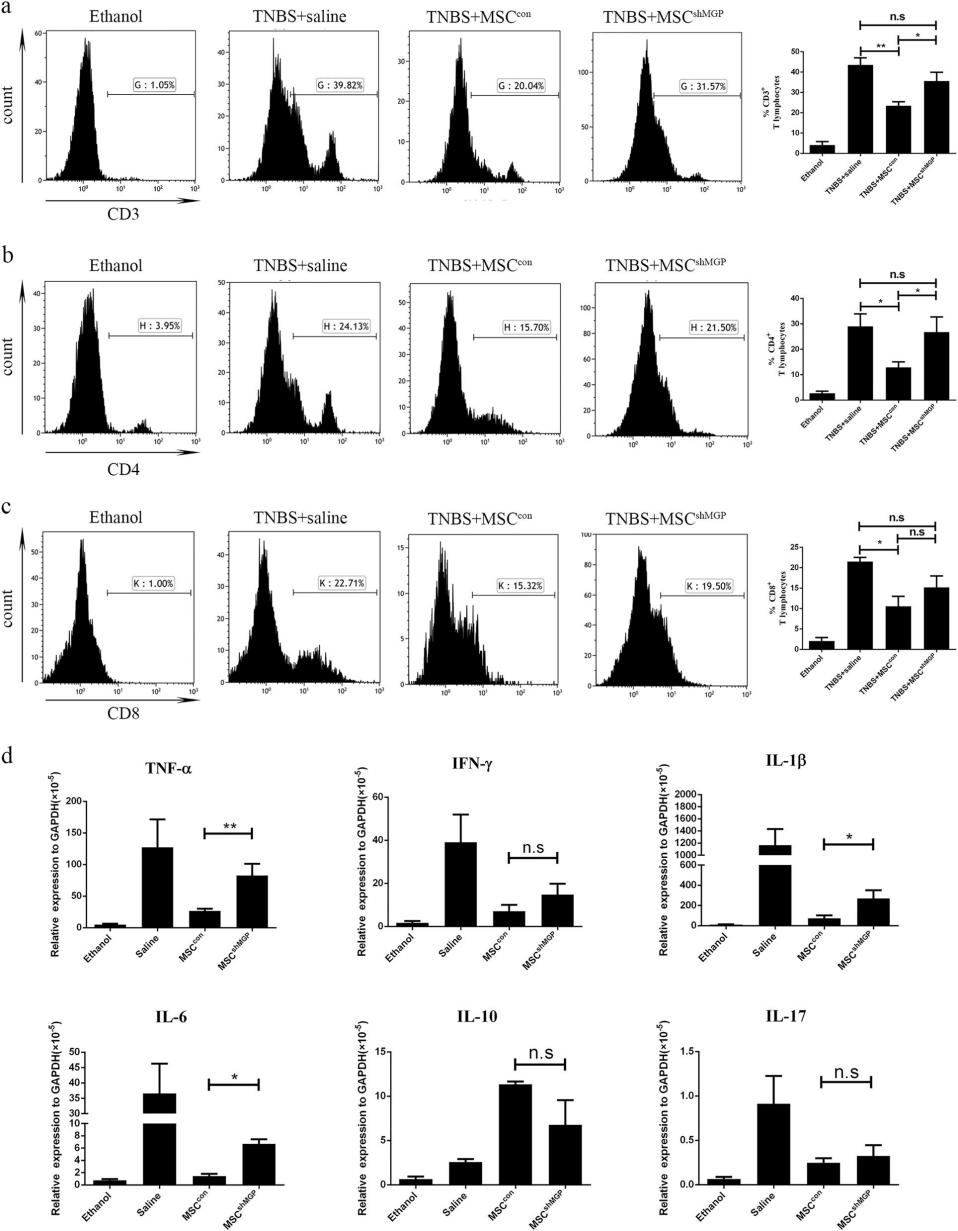
Mouse MSCs suppress the proportion of T cells and pro-inflammatory cytokine expression in local colonic analysis through MGP in an experimental mouse model of colitis. The proportion levels of mouse CD3^+^ T cells (**a**), CD4^+^ T cells (**b**), and CD8^+^ T cells (**c**) were analyzed by flow cytometry. **d** The local colonic expression levels of pro-inflammation cytokines (TNF-α, IFN-γ, IL-1β, IL-6, and IL-17), and IL-10 were analyzed at the mRNA level. Data are shown as mean ± SEM (*n* = 3). *P < 0.05, **P < 0.01, ***P < 0.001, and n.s. means no significant

MSCs have been reported previously to ameliorate TNBS-induced colitis by alleviating the status of inflammation cells infiltrating in the colon^3,29,30^. This study investigated the status of T cells subpopulations infiltration by flow cytometry after colon tissue digestion ex vivo. Specifically, normal mice and the simple 50% alcohol modeling group exhibited less T cells infiltration compared with the TNBS-induced experimental colitis group. The proportion of T cells were significantly reduced in the MSC^con^ group, compared with the MSC^shMGP^ group in CD3^+^ T cells (23.47 ± 3.89% versus 35.62 ± 7.51%, Fig. 5a) and CD4^+^ T cells (12.92 ± 4.84% versus 26.73 ± 10.39%, Fig. 5b), but no significant difference was observed in CD8^+^ T cells (10.56 ± 4.23% versus 15.18 ± 5.67%, Fig. 5c).

We also analyzed the mRNA expression of pro-inflammatory cytokines (including TNF-α, IFN-γ, IL-6, IL-1β, and IL-17), and anti-inflammatory cytokines (IL-10) in tissue-digesting cells. In comparison with the MSC^con^ group, the expression of pro-inflammatory cytokines such as TNF-α, IL-6, and IL-1β increased obviously in the MSC^shMGP^ group (Fig. 5d). Together, these results indicate that MSC-derived MGP may alleviate the status of T cells infiltration and suppress the expression of pro-inflammatory cytokines in colon tissues of TNBS-induced the experimental colitis mouse model.

### In vivo suppressed proportion of T cells as well as decreased expression of pro-inflammatory cytokines in peritoneal lavage fluid

**Fig. 6:**
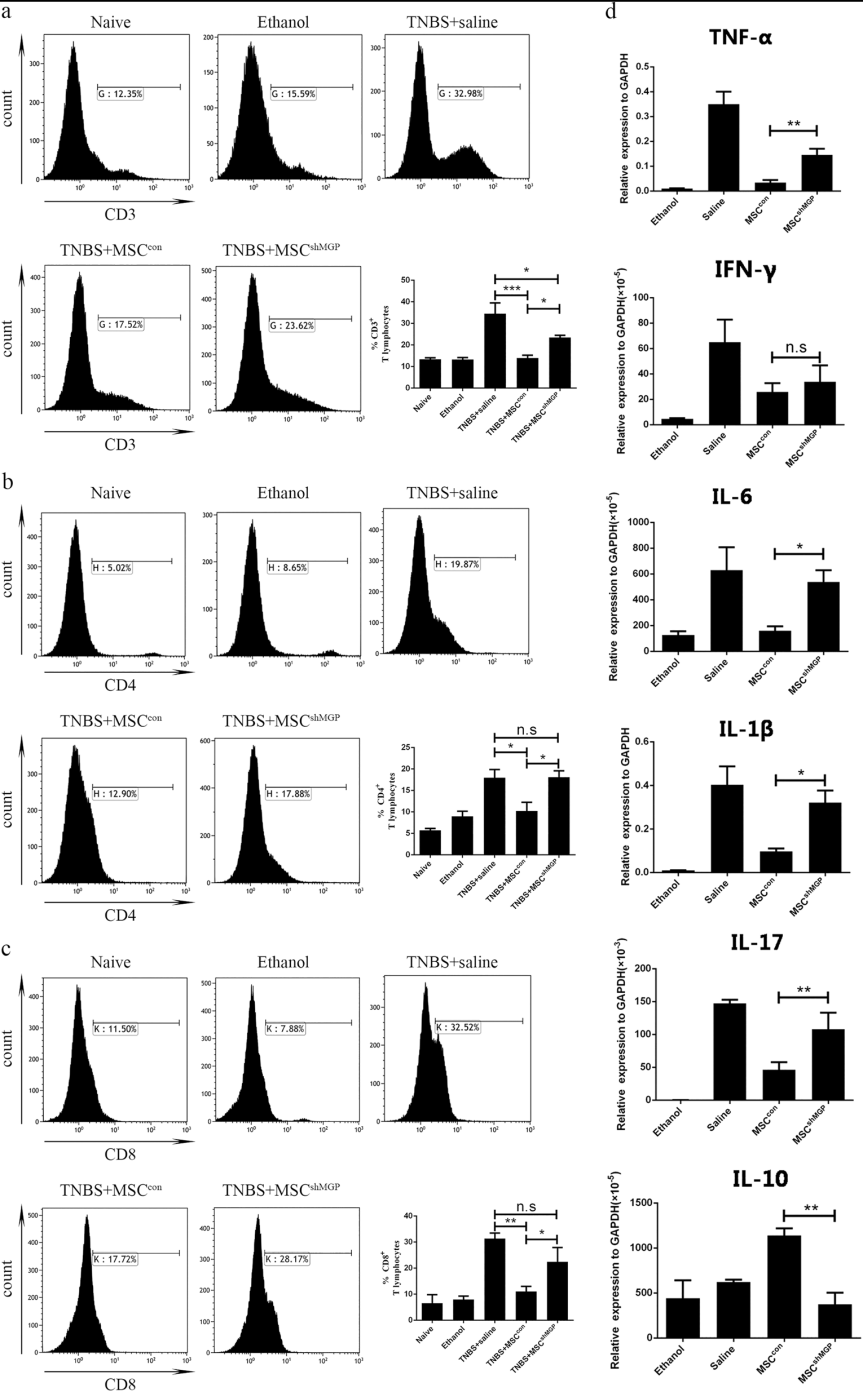
Mouse MSCs suppress the proportion of T cells and pro-inflammatory cytokine expression in intraperitoneal lavage analysis through MGP in an experimental mouse model of colitis. The proportion levels of mouse CD3^+^ T cells (**a**), CD4^+^ T cells (**b**), and CD8^+^ T cells (**c**) were analyzed by flow cytometry. **d** The Intraperitoneal lavage suspension cells expression levels of pro-inflammation cytokines (TNF-α, IFN-γ, IL-1β, IL-6, and IL-17), and IL-10 were analyzed at the mRNA level. Data are shown as mean ± SEM (*n* = 3). *P < 0.05, **P < 0.01, ***P < 0.001, and n.s. means no significant

As CD is primarily associated with the dysfunction of mucosal T cells, we analyzed the proportion of T cells in peritoneal lavage fluid^1^. As expected, normal mice and the simple 50% alcohol modeling group exhibited fewer numbers of lavage cells and a smaller group of T cells compared with TNBS-induced experimental colitis mice. The proportion of T cells was significantly reduced in the MSC^con^ group, compared with the MSC^shMGP^ group in CD3^+^ T cells (13.94 ± 2.67% versus 23.45 ± 2.05%, Fig. 6a), CD4^+^ T cells (10.22 ± 4.10% versus 18.12 ± 2.51%, Fig. 6b), and CD8^+^ T cells (11.06 ± 4.34% versus 22.46 ± 9.49%, Fig. 6c).

We then analyzed the mRNA expression of pro-inflammatory cytokines (including TNF-α, IFN-γ, IL-6, IL-1β, and IL-17), and anti-inflammatory cytokines (IL-10) in lavage cells. In comparison with the MSC^con^ group, the expression of pro-inflammatory cytokines, such as TNF-α, IL-6, IL-1β, and IL-17, increased significantly and IL-10 was decreased in the MSC^shMGP^ group (Fig. 6d).

## Discussion

CD is triggered by an inappropriate and exaggerated intestinal inflammatory response. This response is primarily associated with the dysfunction of mucosal T cells (including activated CD4^+^ Th1 and CD8^+^ CTL cells) and altered cytokine production, that combined, lead to damage of the intestinal mucosa. This study showed the obvious suppression effect of MSCs-derived MGP on proliferation and cytokine production of CD4^+^ and CD8^+^ T cells in vitro, which suggested that MGP might have therapeutic effects on CD. Further experiments in mouse experimental colitis models bore this out. The results showed that MSCs-secreted MGP could ameliorate the clinical and histopathological severity of colonic inflammation, with an obvious inhibiting action on the number of T cells and degree of cytokine production in peritoneal lavage fluid and colon tissues of colitis mice. Downregulation of MGP expression significantly weakened this curative effect.

MGP is a member of the growing family of VKDPs. MGP and OC were the first VKDPs found not to be involved in coagulation and synthesized outside the liver^14^. MGP binds calcium and calcifies matrices through the interaction with their Gla residues^18,31^. Most of the research into MGP has focused on its role in the control of tissue mineralization^19–21,32^, and few studies have hinted that MGP might be associated with inflammation^16,20,22,32,33^. The results of this study have provided evidence to support the immunomodulatory functions of MGP. It has confirmed that MSC-secreted MGP could suppress the proliferation and cytokine production of CD4^+^ and CD8^+^ T cells in vitro and in vivo studies. Few previous studies have investigated the relationship of MGP and cell proliferation/cytokine production. Boström Ket and colleagues reported that human MGP protein could increase (in a dose-dependent manner) VEGF expression and proliferation of endothelial cells from cow aorta^34,35^. Here, we report the detail of how MGP affects immune cells for the first time.

VKDPs are known to be a functional protein family with Gla residues, which result from a γ-carboxylation of glutamate residues, a post translation modification dependent of vitamin K and catalyzed by γ-glutamyl carboxylase^14,36,37^. Previous studies have revealed several members of VKDPs with immunomodulatory properties. For instance, recombinant human-activated PC could protect cells from apoptotic insult, and its ability to suppress nuclear factor-κB suppresses cell activation by thrombin and cytokines, thereby suppressing inflammatory responses^38^. Gas6 and PS display inflammation modulating effects depending on the TAM receptor (Tyro3, Axl, and Mer) types of cells^39^. Gas6 could induce AKT phosphorylation in primary mouse hepatocytes and thus protect them from hypoxia-induced cell death, promote cell growth and survival during tissue repair and development in different organs, and diminish lipopolysaccharide-induced cytokine expression (IL-1β and TNF-α) in murine macrophages^40^. OC is secreted by osteoblasts and improves insulin sensitivity in vivo. Both carboxylated and uncarboxylated forms of OC increased secretion of adiponectin and the anti-inflammatory cytokine IL-10, suppressed secretion of TNF-α. However, only carboxylated OC suppressed IL-6 release, and neither form of OC modulated MCP-1 secretion. POSTN functions as part of the extracellular matrix and a negative-feedback loop regulating allergic inflammation^41^. The carboxylated form of POSTN is produced by bone-derived cells of mesenchymal lineage including MSCs^42^. The mechanism of POSTN's immunomodulatory function may involve augmentation of TGF-β1 and Foxp3-induced Treg cell differentiation^43^. The immunoregulatory functions of MGP added to the growing evidence for the relationship between VKDPs and immunomodulation.

The correlation between Vitamin K and CD have attracted great interests recently. Schoon and colleagues have reported that low serum and bone vitamin K status in patients with longstanding CD^8^. It was consistent with a role for vitamin K deficiency in the pathogenesis of osteoporosis associated with CD^9–12^. Duggan and colleagues showed that vitamin K status of CD patients was lower than that of the healthy controls, suggesting that it might be another etiological factor for CD-related osteopenia^44^. Cravo and colleagues pointed out that low vitamin K intake was more frequent in CD patients. There is adequate evidence to support that vitamin K may play a key role in the progression of CD^45^. Therefore, it seems reasonable that MSCs-derived VKPDs with immunomodulatory function may contribute to CD therapy.

Our data about MGP would be as a proof of this suggestion. As the immunomodulatory ability of MSCs is limited by their heterogeneity and disease microenvironments, in order to stabilize their therapeutic efficacy for CD, it would be an ideal way to gather MSCs subpopulation that high-expressing MGP or VKDPs with immunomodulatory function. This work may offer a new strategy for enriching powerful MSCs to maximize their therapeutic benefits.

## Materials and methods

### Mice

C57BL/6 wild-type mice were purchased from the Animal Center of the Medical Laboratory of Guangdong Province, China. All animals used for in vivo studies were 8-week-old male C57BL/6 mice that were randomly allocated to each group. All animal protocols were reviewed and approved by the Sun Yat-sen University Institutional Animal Care and Use Committee.

### Isolation and culture of mouse MSCs

MSCs were isolated from murine bone marrow by well-established protocols^3^. In brief, femurs and tibias of mice were removed and flushed with l-Dulbecco’s modified essential medium (DMEM) containing 10% (v/v) fetal calf serum (FCS; Hyclone, Logan, UT). Bone marrow cells were obtained by filtration with a 70-μm cell strainer. After red blood cells were removed by ammonium chloride lysis, the remaining cells were washed with Hanks balanced salt solution, added to culture flasks at the low density of 5 × 10^4^ cells/cm^2^ in l-DMEM with 10% (v/v) FCS. The cells were then cultured for 3 days, and non-adherent cells were removed by a complete change of the medium, whereas the remaining adherent cells were cultured continuously.

### RNA isolation and RT-qPCR

RNA was isolated from cells using TRIzol (Invitrogen, Carlsbad, CA) according to manufacturer’s instructions and quantified using a spectrophotometer (NanoDrop). Complementary DNA (cDNA) was prepared using a RevertAid First Strand cDNA Synthesis Kit (Thermo Scientific, Vilnius, Lithuania). Intestinal tissue was homogenized using a TissueLyser (QIAGEN, Valencia, CA). RNA was extracted using Trizol and reverse transcribed. The cDNA thus obtained was subjected to real-time PCR with the SYBR Green reagent (Roche, Indianapolis, IN) using the mouse primers listed in Supplementary Table S1. Expression levels were normalized to those of Glyceraldehyde 3-phosphate dehydrogenase.

### Western blotting analysis

Cells were extracted and the protein concentration was measured using a BCA protein assay kit (Thermo Scientific, Rockford, AL). Proteins were separated using 8% or 12% sulfate-polyacrylamide gel electrophoresis and then transferred to a polyvinylidene fluoride membrane; the membrane was then blocked with Tris-buffered saline/T containing 5% nonfat dry milk and analyzed for the target proteins. The specific antibodies used recognized MGP (ab192396, Abcam, UK).

### Immunofluorescencent staining

MSCs were washed with PBS before being fixed with 1 h treatment of 4% (wt/vol) formalin. Fixed cells were washed twice before being treated with primary antibodies incubated in PBS or permeabilization buffer overnight at 4°C. Following primary antibodies incubations, all cells were washed twice and treated with secondary antibodies for 1 h at room temperature. The secondary antibodies were dissolved in PBS. Cells were then washed twice before being mounted using Fluoroshield with DAPI and glass coverslips. Cells were imaged using a Zeiss Observer Fluorescence Microscope and Axiovision imaging software.

### Construction of the lentivector for RNA silencing

The shRNA was designed in-house and synthesized by Sangon Biotech (Shanghai, China). The sequence is presented in Table S2. The lentiviral vector, LentiLox 3.7(pLL3.7), was used for long-term interference with mouse MSCs. The negative control (designated “con”) was an insert-free vector.

### Construction of the Cas9/sgRNA lentivectors and generation of MGP-knockout MSCs

Lentiviral vectors (LVs) containing (carrying) Cas9 and sgRNA were constructed for transfection of the MSCs. The LVs harboring Cas9 were transfected into MSCs using polybrene, and the MSC‑Cas9 stable cells were selected by puromycin after 48 h of transfection. In addition, two different sgRNAs were designed according to the two different target sites in the MGP gene and packaged into the LVs. The sgRNA was designed in-house and synthesized by Sangon Biotech (Shanghai, China). The sequence is presented in Table S3.

### Mouse MSCs proliferation assay

MSCs were resuspended in l-DMEM with 10% (v/v) FCS and seeded to a 12-well plate at 5 × 10^4^ cells per well. The cells were trypsinised at each indicated time point over 7 days, and cell numbers were counted directly. Simultaneously, MSCs were resuspended in l-DMEM with 10% (v/v) FCS and seeded to a 96-well plate at 3 × 10^3^ cells per well. Medium was changed 24 h later, and 100 μL medium containing 10 μL CCK-8 was added to each well. After 4 h’ culture, the absorbance of each well at 450 nm was measured, MSCs without transfection were used as control.

### Mouse MSCs apoptosis assay

MSCs were resuspended in L-DMEM with 10% (v/v) FCS and seeded to a six-well plate at 1 × 10^5^ cells per well. The cells were trypsinised after 3 days of culture. The percentage of apoptotic MSCs was evaluated using a fluorescein isothiocyanate Annexin V Apoptosis Detection Kit I (BD Pharmingen) according to the manufacturer’s instructions. On the other hand, MSCs were processed a cell staining to assess cell viability using the dye exclusion test by Trypan Blue Solution (Gibco) according to the manufacturer’s instructions.

### Cell surface staining

Flow cytometric analyses were performed with Influx (BD Bioscience, San Jose, CA) or Gallios (Beckman Coulter, Fullerton, CA) flow cytometers, and the data were analyzed with the FlowJo7.5 (Treestar, Ashland, OR) or Kaluza (Beckman Coulter) software packages. Anti-mouse Sca-1-APC (D7), CD106-eFluor 660 (429), CD44-PE (IM7), c-Kit-APCeFluor 780 (2B8), CD140a-PE-Cyanine7 (APA5), CD34-eFluor 660 (RAM34), CD45-PE-Cyanine7 (30-F11), and CD31-APC (390) antibodies which, along with the corresponding isotype control antibodies, were purchased from eBioscience (San Diego, CA). CD3e-PE-CyTM7 (145-2C11), CD4-APC (RM4-5), and CD8a-Pacific Blue TM (53–6.7) were purchased from BD Pharmingen. Propidiumiodide (PI; BD Pharmingen, San Jose, CA) was used to stain dead cells.

### Differentiation assays

For osteogenic and adipogenic differentiations of MSCs in vitro, we used the StemPro™ Osteogenesis Differentiation Kit and StemPro™ Adipogenesis Differentiation Kit, purchased from Thermo Fisher Scientific and used as per manufacturer’s protocol.

### MSC/T cells co-culture assay

Mouse MSCs (2.5 × 10^4^ cells) were plated to a 24-well plate (Corning) and cultured for 48 h. MSCs-derived supernatant after 72 h culture was prepared as conditioned medium (MSC-CM). Mouse splenocytes were washed twice with phosphate-buffered saline containing 3% FCS and then incubated with an anti-mouse CD3 antibody (BD Pharmingen) at 4°C for 30 minutes. Pure CD3^+^ T cells were sorted by flow cytometry (Influx), and 2.5 × 10^6^ T cells were added to each well at a final concentration of 2.5 × 10^6^ T cells per mL in standard Roswell Park Memorial Institute 1640 medium (1 × RPMI with 10 mg/mL glycine, 100 U/mL penicillin, 100 U/mL streptomycin, and 10% (vol/vol) FBS). For T cells proliferation assays, 5,6-carboxyfluorescein diacetatesuccinimidyl ester (CFSE; Invitrogen) staining (5μmol/L) was used. To activate T cells, anti-CD3 and anti-CD28 (BD Pharmingen; final concentration, 500 ng/mL) were added to T cells cultures. After 3 days of activation in the presence of MSCs or MSC-CM, the CD3^+^ T cells were collected and analyzed by flow cytometry. To investigate the ability of MSCs to inhibit the CD4^+^ and CD8^+^ T-cell subpopulations, the collected cells were also stained with an anti-CD4 and -CD8 antibody (eBioscience).

### Apoptosis detection and Treg assays of T cells

For T cells apoptosis detection and Treg assays, we used the methods described in our previous study^3^.

### Intracellular cytokine staining

After 3-day co-cultured with or without MSCs, T cells were stimulated for 6 h with 50 ng/mL phorbol myristate acetate and 0.5 μg/mL ionomycin in the presence of brefeldin A (all from Sigma Aldrich) before collection and analysis. Cells were then washed once and stained with anti-CD4 and anti–CD8 antibodies as described above before fixation with 4% (wt/vol) formalin for 0.5 h. Following fixation, cells were washed twice and then were incubated in permeabilization buffer and stained with anti–IFN-γ and anti–TNF-α antibodies for 30 min at room temperature. After washing twice, T cells were then resuspended in an appropriate volume of washing buffer (300–500 μL) and analyzed by flow cytometry.

### Experimental colitis induced by TNBS

To establish the colitis experimental model, the backs of 8-week-old male BALB/c mice were smeared with 150 μL of pre-sensitization solution (TNBS; Sigma) 7 days before inducing colitis. The mice were divided into six groups (five mice/group) and fasted (but allowed to drink ad libitum) for 24 h. Colitis induction and MSCs treatment was performed as described in our previous study^3^.The parameters of body weight loss, diarrhea, and survival were recorded daily for 7 days. Colons were collected from cecum to the anus 3 days after TNBS injection (the peak of the disease), and the colon length was measured. Colons were evaluated for macroscopic damage according to our previous study^3^.

### Enzyme-linked immunosorbent assay

TNF-α and IFN-γ production was measured using mouse TNF-α and IFN-γ ELISA quantitation kits (all from eBioscience) as per manufacturer’s protocols. For MGP secretion assay, MSCs were seeded in 12-well plate at 5 × 10^4^ cells per well, and MGP in the supernatant was measured using mouse MGP ELISA quantitation kits (Cloud-Clone Corp) as per manufacturer’s protocols.

### The peritoneal lavage fluids analysis

Mice was killed by cervical dislocation and put in a beaker with 70% ethanol before placing in a tissue culture hood. A small incision was made in the abdominal wall, and the skin pulled back to each side. Ten microliter cold PBS was injected into the lower abdominal cavity with a 10 ml needle in order to collect peritoneal lavage fluids. The cell suspension was collected in 15 ml collection tubes and centrifuged before red blood lysis. The cells were then resuspended and analyzed by flow cytometry and RT-qPCR as described above.

### Tissue preparation and detection

Intestinal tissue was obtained from the part of the colon that was inflamed (~ 4 cm from the anus terminus). Tissue samples were cut into small pieces and homogenized on ice in Trizol and analyzed by RT-qPCR as described above.

### Statistical analysis

All results are expressed as mean ± SEM. Statistical comparisons were made using a two-tailed Student’s *t* test (between two groups) or a one-way analysis of variance (for multi-group comparisons). Changes in body weight were compared using a repeated measure analysis of variance. Survival was analyzed using the Kaplan–Meier log-rank test. *P* < 0.05 was considered to represent a significant difference. Analysis and graphing were performed using the Prism 6.01 software package (GraphPad, San Diego, CA).

## Electronic supplementary material

Supplementary material

supplemental figure legends
