# Supplementary material for: Mesenchymal stromal cells-derived matrix Gla protein contribute to the alleviation of experimental colitis
Source: Cell Death Dis. 2018 Jun 7;9(6):691. doi: 10.1038/s41419-018-0734-3 (PMC5992143; doi:10.1038/s41419-018-0734-3)
Supplement: Supplementary file 1 — Supplementary material [file 41419_2018_734_MOESM1_ESM.pdf]

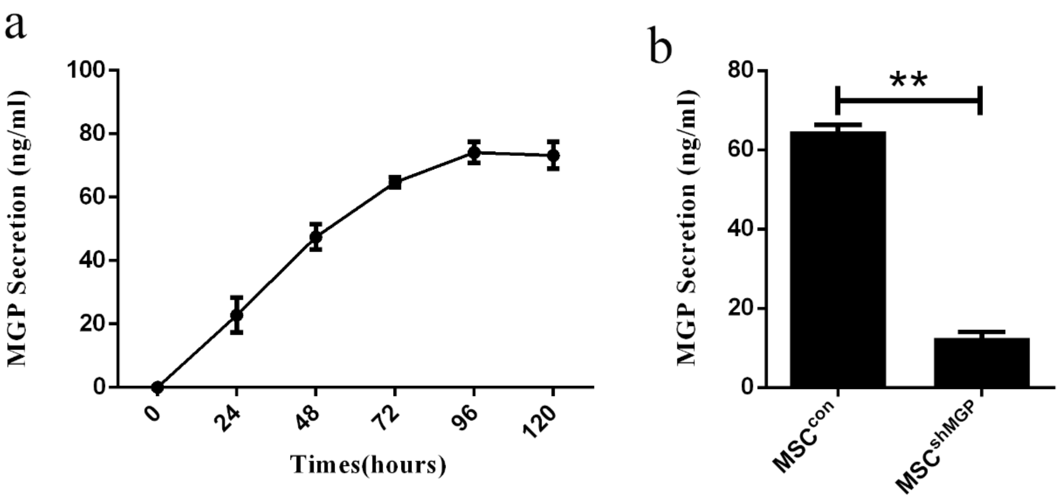

Figure S1. The MGP secretion levels of MSCs were detected by ELISA. (a) The dynamic changes of MSC-secreted MGP among five days. (b) The comparison of MGP secretion between MSC^{con} and MSC^{shMGP} after 72 hours' culture. Data are shown as mean ± SEM (n = 3). **P < 0.01.

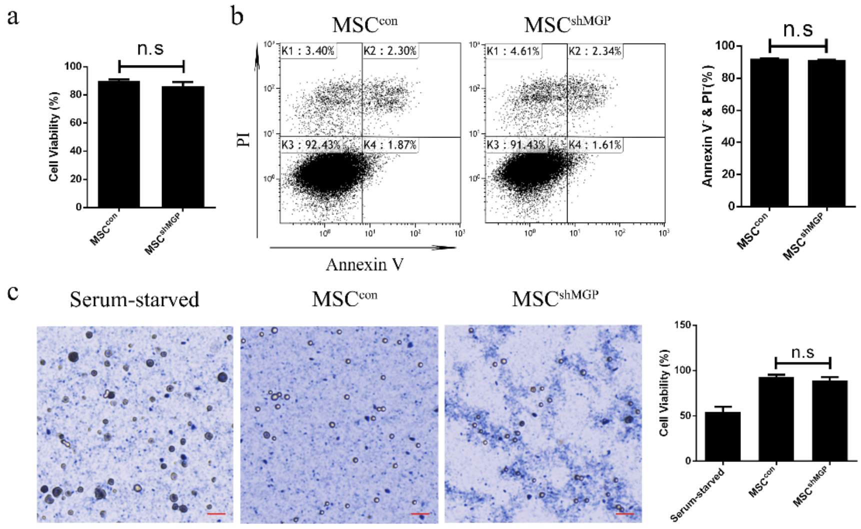

Figure S2. The viability of MSCs was not obviously influenced by the down-regulation of MGP. The proliferation of MSCs was evaluated using the CCK-8 kit (a). The apoptosis of MSCs was evaluated by measuring Annexin V and PI levels (b) and trypan blue staining (c). Cell viability of MSCs were compared using serum-starvation assay by culturing cells without serum for 48h. Data are shown as mean ± SEM (n = 3). Scale bar = 50 µm, and n.s. means no significant.

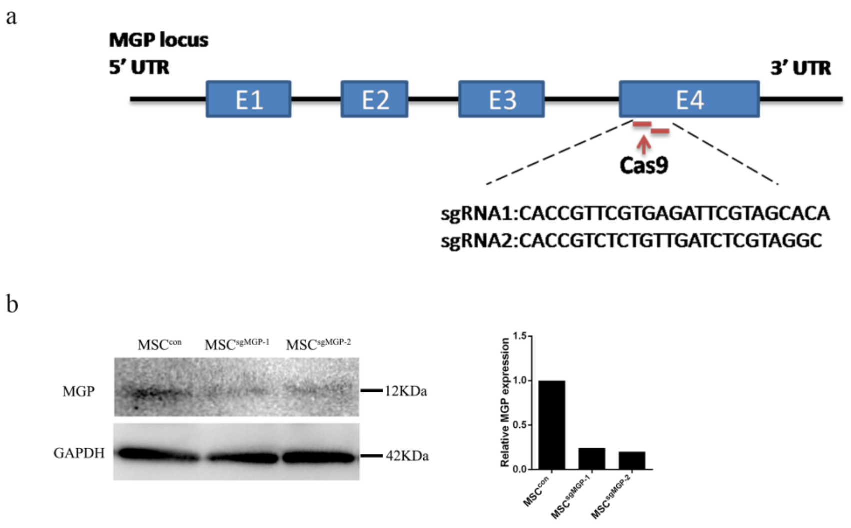

Figure S3. Generation of MGP knockout MSCs. (a) sgRNA/Cas9 was used for long-term MGP knockout in mouse MSCs. (b) The efficiency of sgRNA-mediated down-regulation of MGP was assessed at the protein level. The expression of GAPDH was used as a control.

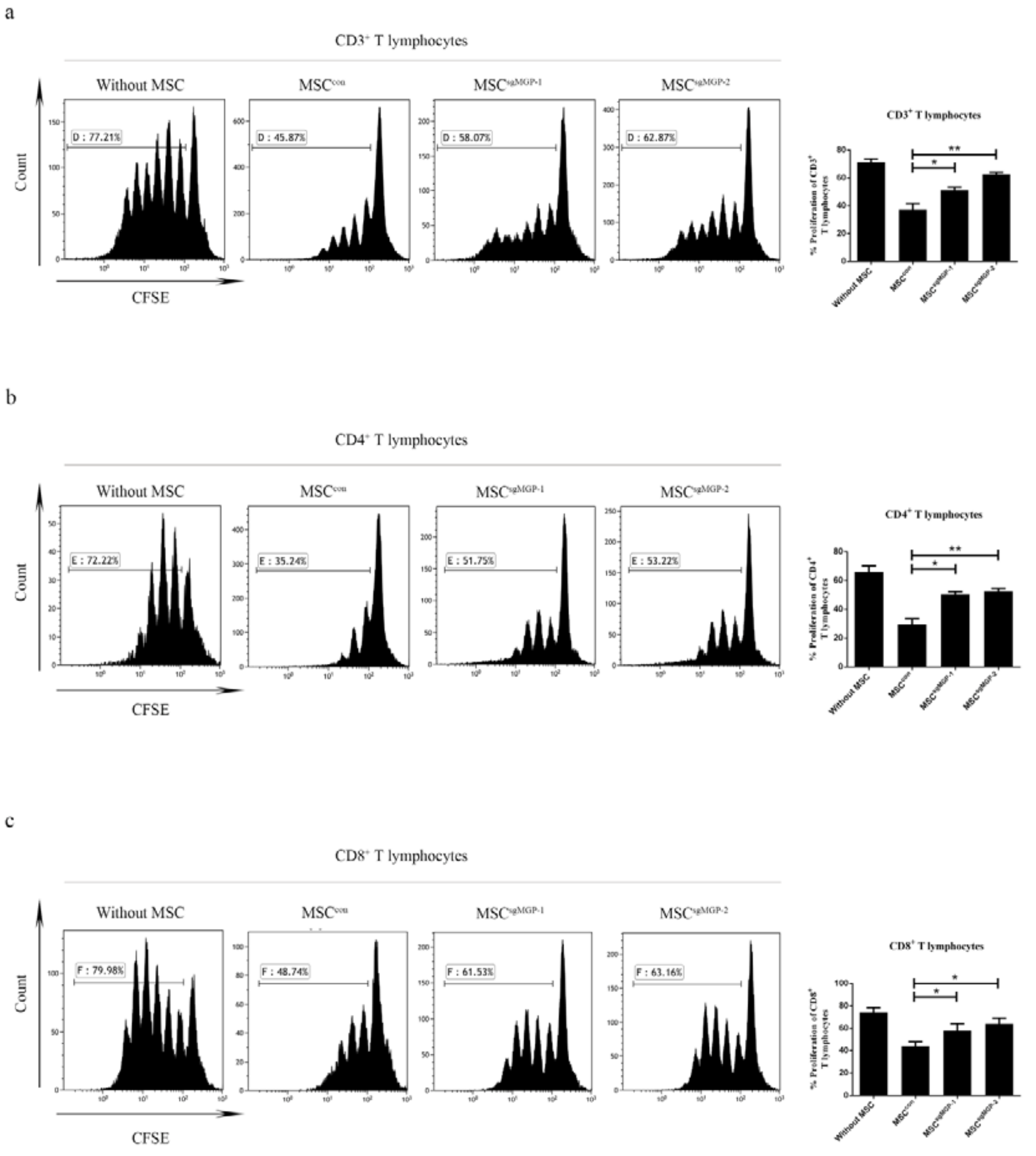

Figure S4. Mouse MSCs-derived MGP inhibits the proliferation of activated T-cells in vitro (verified by CRISPR interference). The proliferation levels of mouse $CD3^+$ T-cells (a), $CD4^+$ T-cells (b) and $CD8^+$ T-cells (c) were analyzed by flow cytometry; the change of CFSE fluorescence intensity indicates the growth ratio. Data are shown as mean ± SEM (n = 3). *P < 0.05, **P < 0.01, ***P < 0.001, and n.s. means no significant.

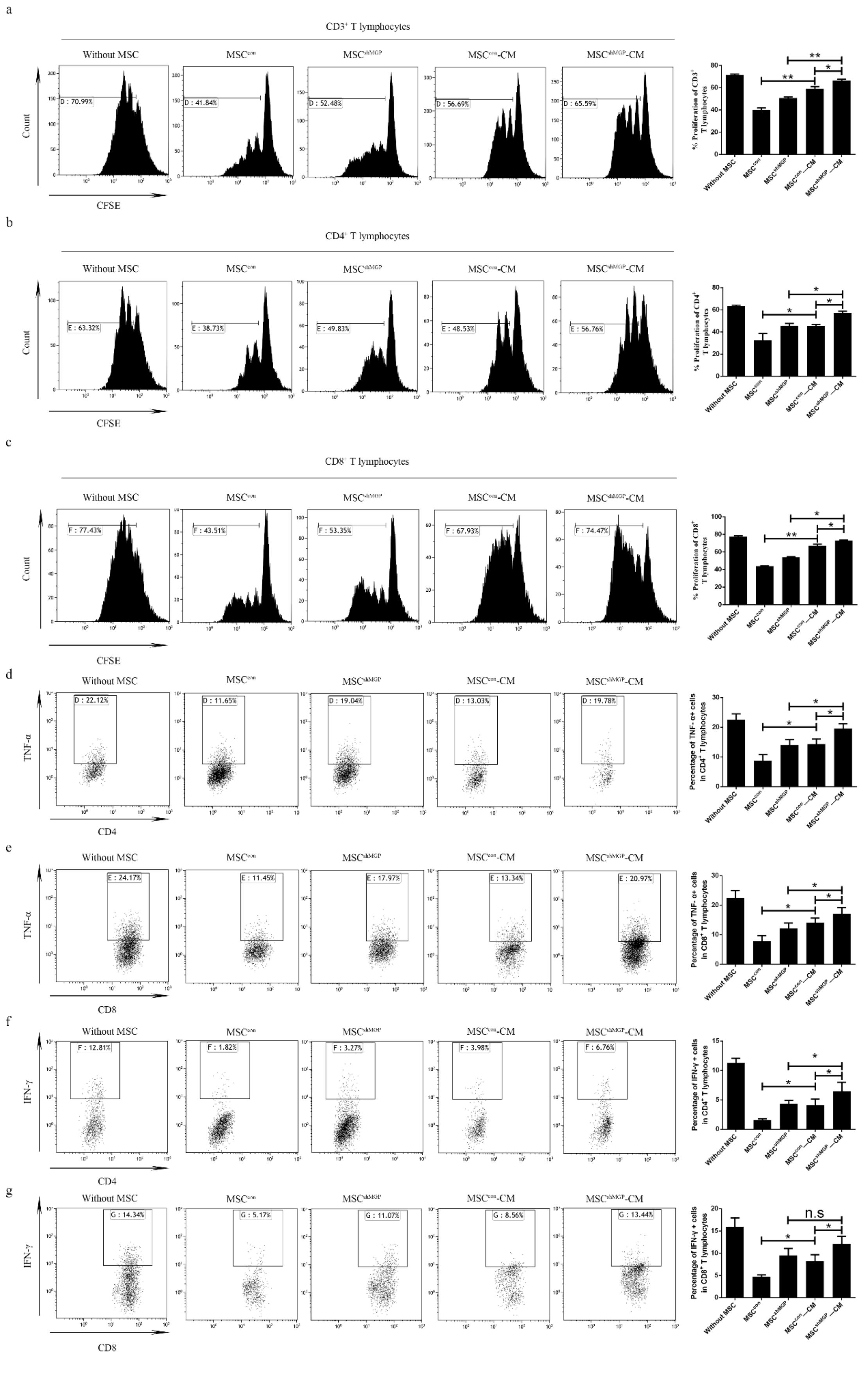

Figure S5. MGP contributes to T-cells immunoregulation of MSCs through a

paracrine manner. The proliferation levels of mouse $CD3^+$ T-cells (a), $CD4^+$ T-cells (b) and $CD8^+$ T-cells (c) were analyzed by flow cytometry; the change of CFSE fluorescence intensity indicates the growth ratio. Flow cytometry was used to analyze the expression levels of TNF-α and IFN-γ in $CD4^+$ T-cells (d and f, respectively) and $CD8^+$ T-cells (e and g, respectively) after 3 days of co-culture with MSCs or MSCs-CM only. Data are shown as mean ± SEM (n = 5). *$P < 0.05$, **$P < 0.01$, and n.s. means no significant.

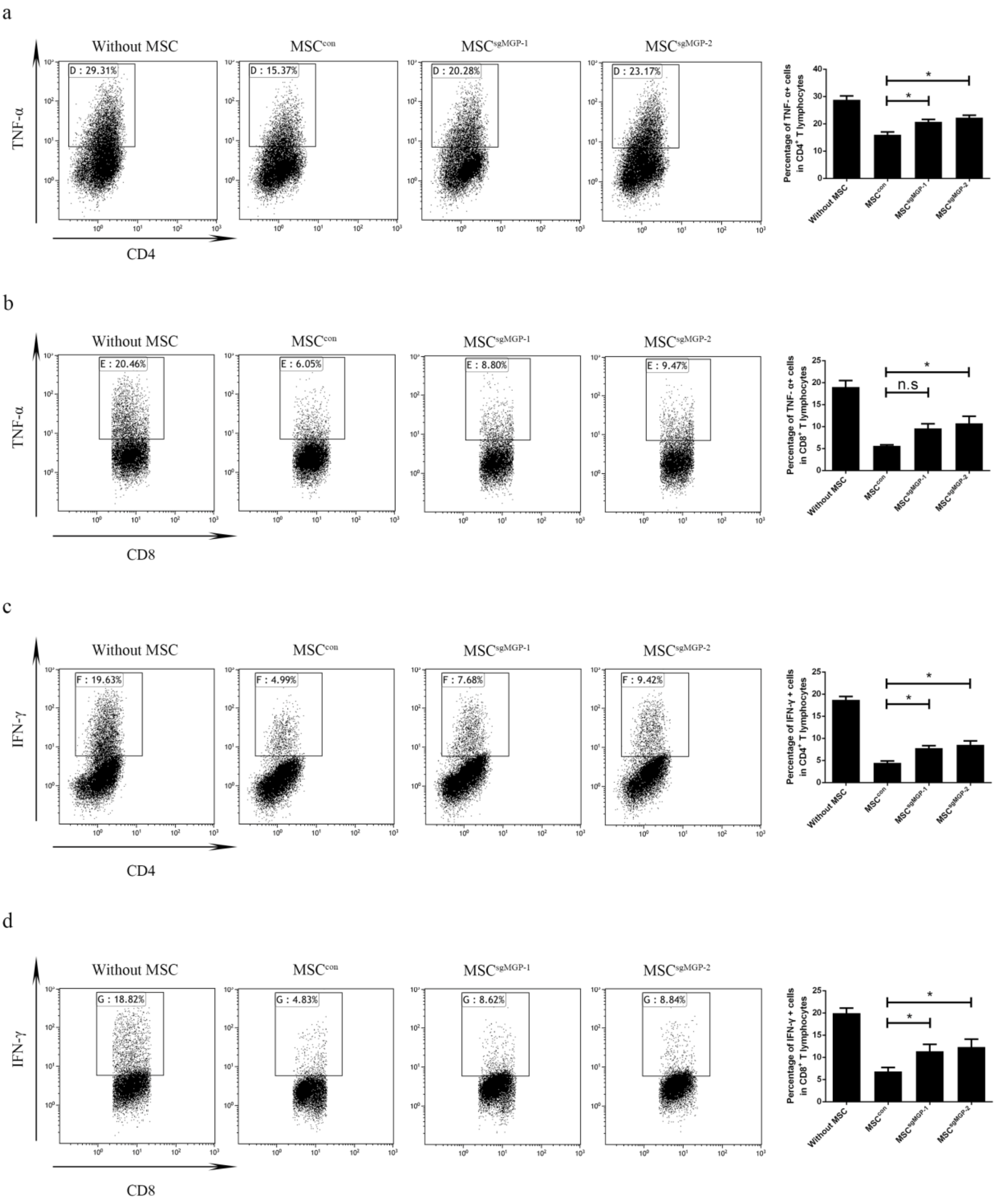

Figure S6. Mouse MSCs-derived MGP down-regulates the cytokine production of activated T-cells (verified by CRISPR interference). Flow cytometry was applied to analyze the expression levels of TNF-α and IFN-γ in $CD4^+$ T-cells (a and c, respectively) and $CD8^+$ T-cells (b and d, respectively) after 3 days of co-culture with MSCs. Data are shown as mean ± SEM (n = 3). *P < 0.05, and n.s. means no significant.

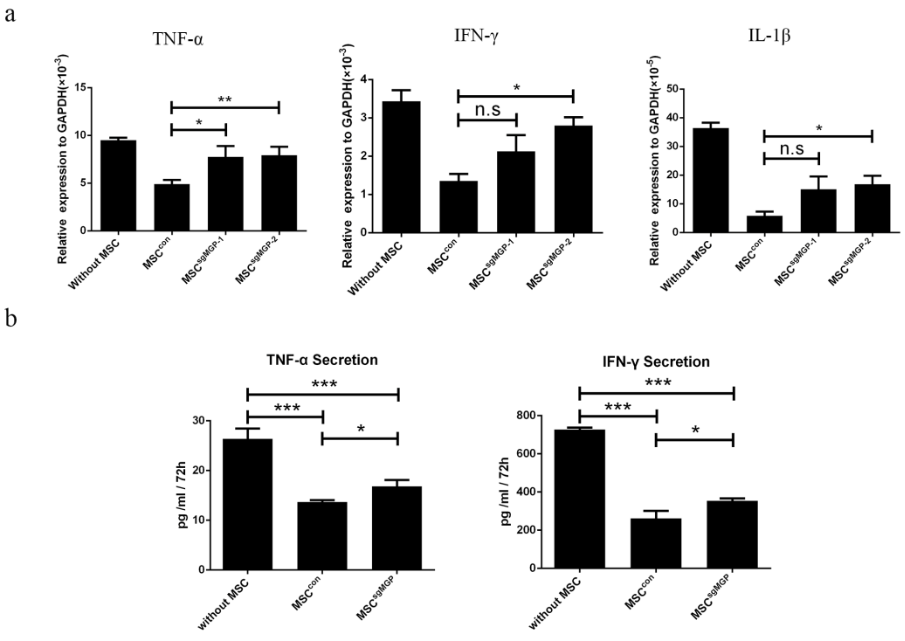

Figure S7. MSCs (MSC^{con} and MSC^{sgMGP}) suppress the cytokine expression and secretion via MGP. (a) The expression levels of pro-inflammation cytokines (TNF-α, IFN-γ and IL-1β) were analyzed at the mRNA level. (b) The secretion levels of pro-inflammation cytokines (TNF-α and IFN-γ) were analyzed by ELISA. Data are shown as mean ± SEM (n = 3). *P < 0.05, **P < 0.01, ***P < 0.001, and n.s. means no significant.

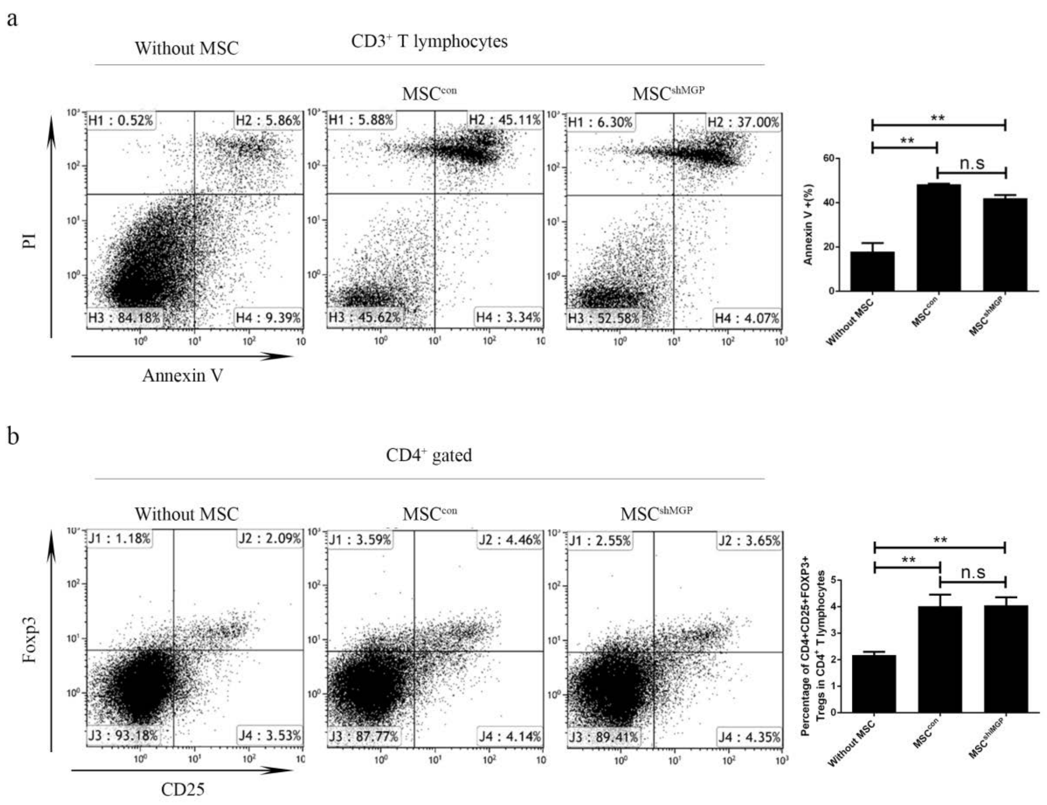

Figure S8. MSCs do not influence the apoptosis of activated T-cells and the differentiation of CD4$^+$CD25$^+$FoxP3$^+$Tregs through MGP. (a) After 3 days of co-culture with or without MSCs, CD3$^+$ T-cells were analyzed for apoptosis using flow cytometry. (b)The proportion of the Treg was analysed by flow cytometry 2 days after T-cells co-culture with MSCs. Data are shown as mean ± SEM (n = 3). **P < 0.01, and n.s. means no significant.

Table S1. Primers used for the amplification of mouse transcripts by qPCR

Genes	Forward sequence	Reverse sequence
GAPDH	5’-ACCACAGTCCATGCCATCAC-3’	5’-TCCACCACCCTGTTGCTGTA-3’
MGP	5’-AGGAACGCAACAAGCCTGC CTA-3’	5’-CTGCCTGAAGTAGCGGTTG TAG-3’
TNF-α	5’-GGTGCCTATGTCTCAGCCT CTT-3’	5’-GCCATAGAACTGATGAGAGG GAG-3’
IL-6	5’-TACCACTTCACAAGTCGGA GGC-3’	5’-CTGCAAGTGCATCATCGTTG TTC-3’
IL-1β	5’-TGGACCTTCCAGGATGAGG ACA-3’	5’-GTTCATCTCGGAGCCTGTA GTG-3’
IL-10	5’-CGGGAAGACAATAACTGCA CCC-3’	5’-CGGTTAGCAGTATGTTGTCC AGC-3’
IL-17	5’-CAGACTACCTCAACCGTTC CAC-3’	5’-TCCAGCTTTCCCTCCGCAT TGA-3’

Table S2. MGP shRNA sequence used to generate lentivirus plasmids for RNA silencing

	Oligonucleotide (5'to3')
Forward	TGGAGAAATGCCAACACCTTCTTCCTGTCAAAGGTGTTGGCATTTCTCCTTTTTTC
Reverse	TCGAGAAAAAAGGAGAAATGCCAACACCTTTGACAGGAAGAAGGTGTTGGCATTTCTC

	CA

Table S3. MGP sgRNA sequence used to generate lentivirus plasmids for gene silencing

		Oligonucleotide (5'to3')
sg1	Forward	CACCGTTCGTGAGATTCGTAGCACA
	Reverse	AAACTGTGCTACGAATCTCACGAAC
sg2	Forward	CACCGTCTCTGTTGATCTCGTAGGC
	Reverse	AAACGCCTACGAGATCAACAGAGAC
