# Supplementary material for: Mesenchymal stromal cells-derived matrix Gla protein contribute to the alleviation of experimental colitis
Source: Cell Death Dis. 2018 Jun 7;9(6):691. doi: 10.1038/s41419-018-0734-3 (PMC5992143; doi:10.1038/s41419-018-0734-3)
Supplement: Supplementary file 2 — supplemental figure legends [file 41419_2018_734_MOESM2_ESM.docx]

**Supplementary Figure Legends**

**Figure S1. The MGP secretion levels of MSCs were detected by ELISA.** (a) The dynamic changes of MSC-secreted MGP among five days. (b) The comparison of MGP secretion between MSC^con^ and MSC^shMGP^ after 72 hours’ culture. Data are shown as mean ± SEM (n = 3). **P < 0.01.

**Figure S2.** **The viability of MSCs was not obviously influenced by the down-regulation of MGP.** The proliferation of MSCs was evaluated using the CCK-8 kit (a). The apoptosis of MSCs was evaluated by measuring Annexin V and PI levels (b) and trypan blue staining (c). Cell viability of MSCs were compared using serum-starvation assay by culturing cells without serum for 48h. Data are shown as mean ± SEM (n = 3). Scale bar = 50 μm, and n.s. means no significant.

**Figure S3. Generation of MGP knockout MSCs.** (a) sgRNA/Cas9 was used for long-term MGP knockout in mouse MSCs. (b) The efficiency of sgRNA-mediated down-regulation of MGP was assessed at the protein level. The expression of GAPDH was used as a control.

**Figure S4. Mouse MSCs-derived MGP inhibits the proliferation of activated T-cells in vitro (****verified by CRISPR interference).** The proliferation levels of mouse CD3^+^ T-cells (a), CD4^+^ T-cells (b) and CD8^+^ T-cells (c) were analyzed by flow cytometry; the change of CFSE fluorescence intensity indicates the growth ratio. Data are shown as mean ± SEM (n = 3). *P < 0.05, **P < 0.01, ***P < 0.001, and n.s. means no significant.

**Figure S5. MGP contributes to T-cells immunoregulation of MSCs through a paracrine manner.** The proliferation levels of mouse CD3^+^ T-cells (a), CD4^+^ T-cells (b) and CD8^+^ T-cells (c) were analyzed by flow cytometry; the change of CFSE fluorescence intensity indicates the growth ratio. Flow cytometry was used to analyze the expression levels of TNF-α and IFN-γ in CD4^+^ T-cells (d and f, respectively) and CD8^+^ T-cells (e and g, respectively) after 3 days of co-culture with MSCs or MSCs-CM only. Data are shown as mean ± SEM (n = 5). *P < 0.05, **P < 0.01, and n.s. means no significant.

**Figure S6. Mouse MSCs-derived MGP down-regulates the cytokine production of activated T-cells (verified by CRISPR interference).** Flow cytometry was applied to analyze the expression levels of TNF-α and IFN-γ in CD4^+^ T-cells (a and c, respectively) and CD8^+^ T-cells (b and d, respectively) after 3 days of co-culture with MSCs. Data are shown as mean ± SEM (n = 3). *P < 0.05, and n.s. means no significant.

**Figure S7. MSCs (MSC^con^ and MSC^sgMGP^) suppress the cytokine expression and secretion via MGP.** (a) The expression levels of pro-inflammation cytokines (TNF-α, IFN-γ and IL-1β) were analyzed at the mRNA level. (b) The secretion levels of pro-inflammation cytokines (TNF-α and IFN-γ) were analyzed by ELISA. Data are shown as mean ± SEM (n = 3). *P < 0.05, **P < 0.01, ***P < 0.001, and n.s. means no significant.

**Figure S8. MSCs do not influence the apoptosis of activated T-cells and the differentiation of CD4^+^CD25^+^FoxP3^+^Tregs through MGP.** (a) After 3 days of co-culture with or without MSCs, CD3^+^ T-cells were analyzed for apoptosis using flow cytometry. (b)The proportion of the Treg was analysed by flow cytometry 2 days after T-cells co-culture with MSCs. Data are shown as mean ± SEM (n = 3). **P < 0.01, and n.s. means no significant.

**Table S1.** Primers used for the amplification of mouse transcripts by qPCR.

**Table S2.** MGP shRNA sequence used to generate lentivirus plasmids for RNA silencing.

**Table S3.** MGP sgRNA sequence used to generate lentivirus plasmids for gene silencing.
